# Characteristics of renal pathology and coagulation function in IgA nephropathy and IgA vasculitis associated nephritis

**DOI:** 10.1186/s12882-024-03465-6

**Published:** 2024-01-25

**Authors:** Yinhong Wang, Hao Wang, Xiaotao Ma, Zikun Zhu, Xuefei Tian, Rongguo Fu, Lining Jia

**Affiliations:** 1 Department of Nephropathy, the Second Affiliated Hospital of Xi’an Jiaotong University, Xi’an, China; 2 Department of Nephropathy, Xi’an No 3 Hospital, the Affiliated Hospital of Northwest University, Xi’an, China; 3 Department of Computer Science, School of Computing & Department of Electrical and Computer Engineering, National University of Singapore, Singapore, Singapore; 4 Section of Nephrology, Department of Internal medicine, Yale University School of Medicine, New Haven, CT USA

**Keywords:** IgA nephropathy, IgA vasculitis associated nephritis, Coagulation, Fibrinogen degradation products

## Abstract

**Background:**

The objective of this study is to investigate the clinical and pathological differences between patients with IgA nephropathy (IgAN) and IgA vasculitis associated nephritis (IgAVN).

**Methods:**

A total of 253 patients with IgAN and 71 patients with IgAVN were retrospectively included in the study, and clinical and laboratory data were collected and analysed.

**Results:**

Compared with IgAVN group, months from onset to kidney biopsy were significantly prolonged in IgAN patients because of the lack of obvious symptoms such as rash, abdominal symptoms, and joint pain (13.5 ± 26.6 vs. 10.2 ± 31.6 months, *P* = 0.007), and the levels of serum creatinine (92.3 ± 94.7 vs. 68.9 ± 69.2 µmol/L, *P* = 0.015) was higher and eGFR (99.1 ± 35.2 vs. 123.4 ± 41.8 mL/min/1.73m^2^, *P* < 0.001) was lower in IgAN group. The pathological results revealed that patients with IgAN have a greater degree of chronic kidney injury compared to patients with IgAVN. In addition, the levels of plasma D-Dimers (1415.92 ± 1774.69 vs. 496.78 ± 711.91 ng/mL, *P* < 0.001) and fibrinogen degradation products (FDP) (3.92 ± 4.73 vs. 1.63 ± 2.46 µg/mL, *P* = 0.001) were significantly higher in IgAVN patients than in IgAN patients. The deposition of fibrinogen in the renal tissues was more severe and the cumulative partial remission rate was higher in patients with IgAVN as compared to those with IgAN (*P* = 0.001).

**Conclusions:**

In comparison, IgAN patients had poorer renal function, whereas IgAVN patients had more severe coagulation abnormalities. These findings provide a basis for the differentiation of the two diseases at an early stage.

**Supplementary Information:**

The online version contains supplementary material available at 10.1186/s12882-024-03465-6.

## Introduction

IgAN is the most common primary glomerulonephritis worldwide and mainly affects the young population [1]. Approximately 30–40% of patients develop end-stage kidney disease within 20–30 years after their initial clinical presentation [2]. IgAVN is a systemic inflammatory disease that affects small blood vessels and is particularly prevalent among children [3]. However, the relationship between IgAN and IgAVN is controversial. Galactose-deficient IgA1, an important effector molecule in IgAN, has been specifically detected in the glomeruli of IgAVN, but not in the other kidney diseases such as lupus nephritis, HCV-related nephropathy, and membranous nephropathy [4]. In addition, the immune transcriptomes of kidney tissue in IgAN, IgAVN, and IgA-dominant infection-related glomerulonephritis largely overlap [5]. IgAVN shares many pathogenetic features with IgAN, which makes it difficult to distinguish IgAVN from IgAN by pathological characteristic alone. Moreover, renal biopsy, being an invasive examination, is unsuitable for patients with solitary kidney and hydronephrosis, and could lead to complications such as renal bleeding and infection. Therefore, the differences in clinical manifestations and laboratory tests may provide some clues for the diagnosis of the two diseases. Recent studies have indicated that coagulation is involved in the pathogenesis of IgAN and IgAVN [6, 7]. Nevertheless, no studies have compared the discrepancies in the coagulation factors levels between the two groups and explored the potential mechanisms. Therefore, in this study, we retrospectively collected and analyzed the clinical, laboratory, coagulation and pathological data of 253 IgAN patients and 71 IgAVN patients, in order to provide useful basis for the early differentiation of the two diseases.

## Methods

### Ethical standards

This study was approved by the ethics committee of the Second Affiliated Hospital of Xi’an Jiaotong University and conducted according to the declaration of Helsinki. All study participants were informed of the study’s purpose, and written informed consent was obtained from the patients or their legal representatives.

### Inclusion and exclusion criteria

Patients with IgAN and IgAVN who underwent kidney biopsy in the Second Affiliated Hospital of Xi’an Jiaotong University from January 2017 to June 2020 were enrolled in the study. The diagnostic criteria for IgAN: kidney biopsy confirmed that predominant mesangial deposition of IgA, those with secondary causes of mesangial IgA deposits were excluded. The diagnostic criteria for IgAVN meets EULAR/PRINTO/PRES diagnostic criteria [8].

Inclusion criteria were: (1) age 18–70 years; (2) patients with newly diagnosed, biopsy-proven IgAN; (3) patients with renal involvement and the presence of purpura or petechiae predominantly in the lower limbs were diagnosed with IgAVN. Exclusion criteria were: (1) Crohn’s disease, ulcerative colitis, ankylosing spondylitis; (2) Chronic mucosal infections (streptococcus, staphylococcus); (3) HBV, HCV, HIV or Cytomegalovirus infections; (4) autoimmune disorders including ankylosing spondylitis, rheumatoid arthritis, systemic lupus erythematosus, dermatitis herpetiformis, sjögren’s syndrome, psoriasis; (5) malignancies such as IgA myeloma, Non-Hodgkin’s lymphoma, Hodgkin’s lymphoma, cutaneous T-cell lymphoma, lung cancer, renal cell carcinoma; (6) patients receiving anticoagulant or antiplatelets therapy; (7) patients with a history of thromboembolism; (8) thrombocytopenic purpura; (9) ANCA-associated vasculitis; (10) incomplete clinical data; (11) pregnancy or lactation.

Pathology and scoring methods.

The pathological specimens in the study were reviewed independently by two experienced renal pathologists. The histological lesions were scored referring to the study by Jiang L et al. [9], and the glomerular, tubular, interstitial and vascular lesions were scored separately. In addition, pathological data for IgAN and IgAVN were scored using the updated Oxford Classification criteria. The semiquantitative histological scoring of immune complexes in renal tissue was assessed in accordance with the research by Lv Y et al. [10]. The grade of deposition of immune complexes was defined as follows: -, negative; 1 +, weak but definite staining; 2 +, moderate staining; 3 +, strong staining; 4 +, bright staining.

Outcome.

The endpoint was the complete remission rate and partial remission rate. Complete remission was defined as proteinuria ≤ 0.3 g/24 h, serum albumin > 35 g/L, and normal serum creatinine level. Partial remission was defined as proteinuria > 0.3 g/24 h, but > 50% decline from baseline, serum albumin level ≥ 35 g/L, and stable serum creatinine [11].

Statistical analysis.

Statistical analysis was performed using SPSS software (version 26.0, IBM Corporation, Armonk, NY). The data of normal or approximately normal distribution were expressed as the means ± standard deviations (SD), and using the Student’s *t*-test to compared the differences between two groups. The qualitative data were expressed as percentages and analysed with Chi-squared Test or Fisher’s exact probability test. The remission rates were analyzed by the Kaplan–Meier method and compared by log-rank test. *P* < 0.05 was considered significant.

## Results

### Clinical characteristics and laboratory parameters of IgAN and IgAVN

As shown in Table 1, there was no significantly difference in age, gender and hypertension between the two groups. Months from onset to kidney biopsy were significantly prolonged in IgAN patients than in IgAVN patients (13.5 ± 26.6 vs. 10.2 ± 31.6 months, *P* = 0.007), which may be due to the lack of obvious symptoms such as rash, abdominal symptoms, and joint pain in IgAN patients.

**Table 1:** Clinical characteristics and laboratory parameters of IgAN and IgAVN patients

	IgAN	IgAVN	P value
Gender (male/ female, n)	119/134	33/38	0.934
Age (years)	35.1 ± 11.9	32.3 ± 15.9	0.106
Hypertension (n, %)	67 (26.5%)	13 (18.3%)	0.158
Rash (n, %)	5 (2%)	71 (100%)	0.001
Abdominal symptoms (n, %)	5 (2%)	14 (19.7%)	0.000
Joint pain (n, %)	0 (0%)	18 (25.4%)	0.000
Oedema (n, %)	44 (17.4%)	4 (5.6%)	0.013
Gross haematuria (n, %)	48 (19.0%)	3 (4.2%)	0.002
Microscopic haematuria (n, %)	234 (93.2%)	67 (94.4%)	0.731
Dysmorphic RBC > 80% (n, %)	226 (94.96%)	60 (92.31%)	0.604
Months from onset to biopsy	13.5 ± 26.6	10.2 ± 31.6	0.007
WBC (*10^^9^/L)	6.5 ± 2.1	8.1 ± 3.0	< 0.001
Hb (g/L)	134 ± 22.2	137.2 ± 19.2	0.110
PLT (*10^^9^/L)	235.3 ± 65.9	223.9 ± 67.0	0.303
Urinary protein (g/24 h)	2.1 ± 0.6	1.997 ± 0.3	0.842
Urinary Kap (mg/L)	35.1 ± 43.9	63.8 ± 157.1	0.023
Urinary Lam (mg/L)	19.5 ± 31.8	35.7 ± 86.3	0.030
TP (g/L)	66.8 ± 9.1	64.4 ± 8.7	0.083
ALB (g/L)	39.5 ± 6.3	38.55 ± 6.5	0.230
Scr (µmol/L)	92.3 ± 94.7	68.9 ± 69.2	0.015
UA (µmol/L)	355 ± 98.6	300.4 ± 90.8	< 0.001
eGFR(mL/min/1.73m^2^)	99.1 ± 35.2	123.4 ± 41.8	< 0.001
IgA (g/L)	3.2 ± 1.3	3.1 ± 1.3	0.508
IgE (IU/mL)	149.3 ± 359.9	79.2 ± 128.4	0.010
Treatment			
ACEIs/ARBs alone (n, %)	69 (33.3%)	5 (7.6%)	< 0.001
Prednisone (n, %)	116 (56%)	52 (78.8%)	
Prednisone + MMF (n, %)	11 (0.53%)	3 (4.5%)	
Prednisone + CTX (n, %)	11 (0.53%)	6 (9.1%)	

IgAN: IgA nephropathy, IgAVN: IgA vasculitis associated nephritis, RBC: red blood cell, WBC: white blood cell, Hb: hemoglobin, PLT: platelet, Kap: kappa light chains, Lam: lambda light chains, TP: total serum protein, ALB: serum albumin, Scr: serum creatinine, eGFR: estimated glomerular filtration rate, UA: serum uric acid; ACEI: angiotensin-converting enzyme inhibitor, ARB: angiotensin receptor blockers, MMF: mycophenolate mofetil, CTX: cyclophosphamide. Categorical variables are presented as the number of patients (percentage). Continuous variables are presented as mean ± standard deviation

Laboratory test results in Table 1 showed that the white blood cell count in the IgAVN group was significantly higher than that in the IgAN group (8.1 ± 3.0 vs. 6.5 ± 2.1 *10^^9^/L, *P* < 0.001), which may be due to the more severe involvement of small vessels in IgAVN. Although there was no difference in urinary protein excretion and serum albumin levels between the two groups, the levels of urinary kappa and lambda light chains in the IgAVN group was significantly higher than that in the IgAN group. Compared with IgAVN group, the levels of serum creatinine (92.3 ± 94.7 vs. 68.9 ± 69.2 µmol/L, *P* = 0.015) was higher and eGFR (99.1 ± 35.2 vs. 123.4 ± 41.8 mL/min/1.73m^2^, *P* < 0.001) was lower in IgAN group.

The treatment regimens of 207 patients with IgAN and 66 patients with IgAVN were collected (Table 1). The results showed that all patients in the study, except those with contraindications, received angiotensin-converting enzyme inhibitors (ACEIs) or angiotensin receptor blockers (ARBs) to reduce urinary protein excretion and control blood pressure. Additionally, the percentage of patients receiving prednisone was 56% in IgAN group and 78% in IgAVN group, respectively.

### Renal pathology of IgAN and IgAVN

As shown in Table 2, renal pathological findings showed that fibrocellular or fibrous crescents, thickness of blood vessel wall, glomerular sclerosis, interstitial inflammatory cell infiltration, interstitial fibrosis and tubular atrophy were more common in IgAN patients (*P* < 0.05), while cellular crescents were more common in IgAVN patients (*P* < 0.05). The activity and chronicity indexes of renal lesions were evaluated. Patients with IgAN exhibited higher chronicity index (2.34 ± 1.81 vs. 1.03 ± 1.24, *P* < 0.001), and total biopsy scores (5.24 ± 2.63 vs. 3.69 ± 2.59, *P* < 0.001) compared to those with IgAVN. The Oxford Classification of IgAN and IgAVN is shown in Supplementary Table 1. The percentages of IgAN patients were 71.1%, 11.9%, 68.8%, 11.5%, 4.3%, 22.5%, and 1.6% for M1, E1, S1, T1, T2, C1, and C2, respectively. In contrast, the percentages of IgAVN patients were 81.7%, 19.7%, 32.4%, 2.8%, 35.2% and 2.8% for M1, E1, S1, T1, C1, and C2, respectively. Compared to the IgAVN group, the IgAN group had a higher proportion of patients for S1 (*P* < 0.001) and T1 + T2 (*P* = 0.002), and a lower proportion for C1 + C2 (*P* = 0.02).

**Table 2:** Pathological characteristics of IgAN and IgAVN patients

	IgAN	IgAVN	P value
Number of glomeruli	19.21 ± 8.93	22.14 ± 9.94	0.007
Cellular crescents (n, %)	58 (23%)	23 (38%)	0.011
Fibrocellular or fibrous crescents (n, %)	174 (68.8%)	28 (39.4%)	< 0.001
Endothelial cell proliferation (n, %)	30 (11.9%)	14 (19.7%)	0.088
Mesangial cell proliferation (n, %)	250 (98.8%)	71(100%)	> 0.999
Glomerular sclerosis (n, %)	155(61.3%)	23(32.4%)	< 0.001
Interstitial inflammatory cell infiltration (n, %)	143(56.5%)	17(23.9%)	< 0.001
Interstitial fibrosis/tubular atrophy (n, %)	134 (53%)	12 (16.9%)	< 0.001
Thickness of blood vessel wall (n, %)	155(61.3%)	20(28.2%)	< 0.001
Activity index	2.27 ± 1.22	2.38 ± 1.56	0.326
Chronicity index	2.34 ± 1.81	1.03 ± 1.24	< 0.001
Total biopsy score	5.24 ± 2.63	3.69 ± 2.59	< 0.001

IgAN: IgA nephropathy, IgAVN: IgA vasculitis associated nephritis. Categorical variables are presented as the number of patients (percentage). Continuous variables are presented as mean ± standard deviation

These pathological findings show that patients with IgAN have a greater degree of chronic kidney injury compared to patients with IgAVN, as evidenced by an increased presence of fibrous crescents and more severe interstitial fibrosis and tubular atrophy, which is consistent with the higher serum creatinine and lower eGFR levels observed in the IgAN group.

### Coagulation parameters of IgAN and IgAVN

We compared coagulation parameters between IgAVN and IgAN patients. The results showed that the levels of plasma D-Dimers (1415.92 ± 1774.69 vs. 496.78 ± 711.91 ng/mL, *P* < 0.001) and FDP (3.92 ± 4.73 vs. 1.63 ± 2.46 µg/mL, *P* = 0.001) were significantly higher in IgAVN patients than in IgAN patients, while the level of APTT (23.82 ± 6.08 vs. 26.93 ± 4.37 s, *P* = 0.007) was significantly lower in IgAVN patients than in IgAN patients (shown in Table 3). There was no significant difference in the levels of prothrombin time (PT), international normalized ratio (INR), fibrinogen, and thrombin time (TT) between the two groups.

**Table 3:** Coagulation parameters of IgAN and IgAVN patients

	IgAN	IgAVN	P value
PT (s)	10.14 ± 0.86	9.87 ± 0.96	0.180
INR	0.92 ± 0.08	0.89 ± 0.09	0.082
APTT (s)	26.93 ± 4.37	23.82 ± 6.08	0.007
FIB (mg/dL)	328.05 ± 104.99	328.46 ± 98.08	0.944
TT (s)	18.94 ± 1.82	18.91 ± 2.10	0.617
D-Dimers (ng/mL)	496.78 ± 711.91	1415.92 ± 1774.69	< 0.001
FDP (µg/mL)	1.63 ± 2.46	3.92 ± 4.73	0.001

IgAN: IgA nephropathy, IgAVN: IgA vasculitis associated nephritis, PT: prothrombin time, INR: international normalized ratio, APTT: activated partial thromboplastin time, FIB: fibrinogen, TT: thrombin time, FDP: fibrinogen degradation products. Continuous variables are presented as mean ± standard deviation

### Immunopathology data of IgAN and IgAVN

In the analysis of the renal immunopathology data of the patients, we did not observe any difference in the levels of IgA, IgG, IgE and C1q deposition in glomeruli between the IgAN and IgAVN groups (shown in Table 4). The percentage of patients with C3 deposition grade 2 + and 3 + was considerably higher in the IgAN group than in the IgAVN group (70.8% vs. 55%, *P* = 0.042), whereas the percentage of patients with fibrinogen deposition grade 2 + and 3 + was markedly lower in the IgAN group than in the IgAVN group (19% vs. 46%, *P* < 0.001).

**Table 4:** Immunopathological data between IgAN and IgAVN patients

Immune complexes	Grade of deposition	IgAN	IgAVN	P value
IgG	-	187(74%)	55(77.5%)	0.343
	1+	52(20.5%)	15(21.1%)	
	2+, 3+	14(5.5%)	1(1.4%)	
IgM	-	17(6.7%)	4(5.6%)	0.877
	1+	161(63.6%)	44(62%)	
	2+, 3+	75(29.6%)	23(32.4%)	
IgA	1+	9(3.6%)	1(1.4%)	0.241
	2+	36(14.2)	5(7%)	
	3+	183(72.3%)	55(77.5%)	
	4+	25(9.9%)	10(14.1%)	
C3	-	12(4.7%)	5(7%)	0.042
	1+	62 (24.5%)	27(38%)	
	2+, 3+	179(70.8%)	39(55%)	
C1q	-	230(90.9%)	66(93%)	0.682
	1+	21(8.3%)	4(5.6%)	
	2+, 3+	2(0.8%)	1(1.4%)	
FIB	-	180(71%)	29(41%)	< 0.001
	1+	26(10%)	9(13)	
	2+, 3+	47(19%)	33(46%)	

IgAN: IgA nephropathy, IgAVN: IgA vasculitis associated nephritis, FIB: fibrinogen. Categorical variables are presented as the number of patients (percentage)

### The remission rates of IgAVN and IgAN

**Fig. 1:**
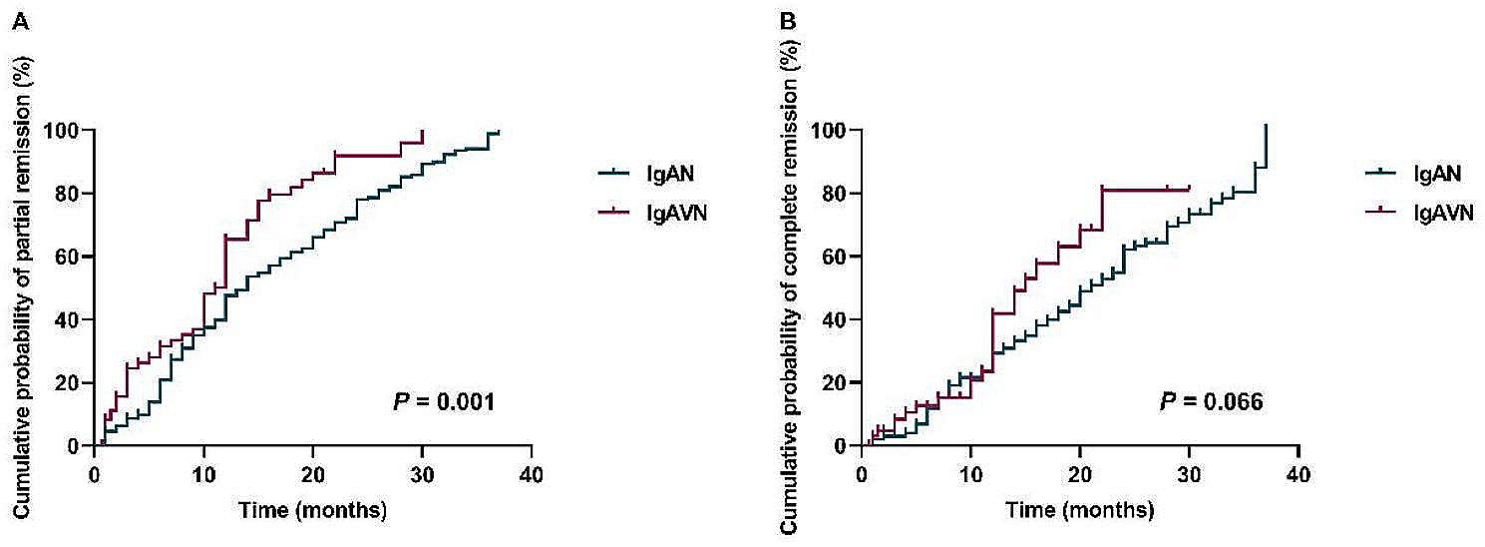
Cumulative probability of partial and complete remission rates in IgAN and IgAVN patients. (**A**) cumulative probability of partial remission rates; (**B**) cumulative probability of complete remission rates. (IgAN: IgA nephropathy, IgAVN: IgA vasculitis associated nephritis)

Kaplan-Meier analysis in Fig. 1 showed that the median time to achieve partial remission was 14 months (95% CI: 10.8–17.1 months) in the IgAN group and 12 months (95% CI: 10.6–13.4 months) in the IgAVN group. The IgAVN group exhibited a higher cumulative partial remission rate compared to the IgAN group (*P* = 0.001). The median time to complete remission was 21 months (95% CI: 18.5–23.4 months) in the IgAN group and 15 months (95% CI: 10.7–19.3 months) in the IgAVN group. However, the log-rank test revealed no significant difference in the cumulative complete remission rates between the two groups.

## Discussion

IgAVN shares many pathogenetic features with IgAN, which poses a challenge in distinguishing between the two diseases by pathological characteristics exclusively. Extra-renal symptoms, such as rash, abdominal symptoms, and joint pain are critical in the early diagnosis of IgAVN. In our study, the time from onset to renal biopsy was significantly longer in patients with IgAN than in patients with IgAVN. This difference may be due to the lack of extrarenal manifestations in patients with IgAN. Renal biopsy is a crucial diagnostic method for evaluating renal injury. In our study, the Oxford Classification of IgAN showed that the percentages of patients were 71.1%, 11.9%, 68.8%, 11.5%, 4.3%, 22.5%, and 1.6% for M1, E1, S1, T1, T2, C1, and C2, respectively. These results are consistent with the study by Coppo, R et al. [12], who revealed that M1, S1 and T1–T2 lesions were predictors of poorer kidney survival. Our study revealed that the chronic lesions, including fibrocellular or fibrous crescents, thickness of blood vessel wall, interstitial fibrosis, and tubular atrophy, were more common in IgAN patients, while cellular crescents were more common in IgAVN patients. Furthermore, the IgAN group exhibited a comparatively higher serum creatinine level and lower eGFR levels than the IgAVN group. Our results indicates that IgAN is a more clandestine disease than IgAVN and often leads to poorer renal function at the initial diagnosis. The extent of tubulointerstitial fibrosis and tubular atrophy is strongly correlated with eGFR and the extent of chronic kidney damage, and is associated with the prognosis of the kidney [13].

These outcomes are in line with the investigation carried out by Lv et al. [10], who analysed data obtained from 809 paediatric patients with IgAVN and 236 paediatric patients with IgAN. Their findings revealed that IgAN patients exhibited more severe renal interstitial injury, fibrous crescents, and other chronic injury manifestations than IgAVN patients. Additionally, remission rate is a crucial indicator for evaluating the prognosis of IgAN and IgAVN. The results in our study indicate that the cumulative partial remission rate was lower in the IgAN group compared to the IgAVN group. This difference may be associated with poorer renal function and more severe interstitial fibrosis and tubular atrophy in IgAN patients.

Coagulation dysfunction is an important non-immune factor in the progression of ESKD [14]. Microvascular endothelial cells injury, activation of platelets, and released plasma coagulation factors can lead to diffuse coagulation in the renal microvasculature, then promotes kidney fibrosis [15]. Study has also shown that heparin may reduce the risk of proteinuria in patients with IgAVN [16].

Plasma D-Dimers formation or elevation reflects activation of the coagulation and fibrinolytic systems. Study have shown that plasma D-Dimer was associated with microalbuminuria [17], and high levels of plasma D-Dimer were associated with the progression of IgAN [18]. In addition, the elevated D-Dimers levels were significantly associated with renal involvement in IgAVN [19]. The results in our study showed that the level of D-Dimers was significantly higher in IgAVN patients than in IgAN patients, which suggests that IgAVN is more active than IgAN [20].

As is well-known, fibrinogen plays an essential function in blood clotting, and in vivo study has confirmed its significant involvement in the promotion of kidney fibrosis [21]. Fibrinogen deficiency protects mice with unilateral ureteral obstruction from interstitial damage, tubular disruption, collagen accumulation, and expression of α-smooth muscle actin in the obstructed kidney. In addition, study of Qin et al. [22] has demonstrated that low albumin-to-fibrinogen (< 12.44) is an independent prognostic factor of poor renal prognosis in Chinese IgAN patients, and the glomerular fibrinogen deposition in IgAVN patients exhibit more severe glomerular damage [23]. In our study, glomerular fibrinogen deposition was observed to be more severe in IgAVN despite no significant difference in plasma fibrinogen levels between the IgAN and IgAVN groups. Plasma FDPs, another crucial factor in the fibrinolytic system strongly linked to IgAVN disease activity [24], were found to be significantly greater in IgAVN patients compared to IgAN patients in our study.

Nevertheless, this study has some limitations that must be acknowledged. Firstly, only patients who underwent kidney biopsies were included in this study. Secondly, it’s unclear whether the patients were in the acute or chronic phase at the time of renal biopsy. Additionally, the small sample size may have an impact on the results obtained. Therefore, it is essential to expand the sample size and monitor patients’ prognoses to establish a theoretical foundation for early diagnosis and treatment.

## Conclusions

In comparison, IgAN patients had poorer renal function, whereas IgAVN patients had more severe coagulation abnormalities. These findings provide a basis for the differentiation of the two diseases at an early stage.

### Electronic supplementary material

Below is the link to the electronic supplementary material.

Supplementary Material 1

## Data Availability

The datasets used and analysed are available from the correasponding author on reasonable request.

## Abbreviations

IgAN: IgA nephropathy
IgAVN: IgA vasculitis associated nephritis
WBC: White blood cell
Hb: hemoglobin
PLT: Platelet, Kap:kappa light chains
Lam: Lambda light chains
TP: Total serum protein
ALB: Serum albumin
Scr: Serum creatinine
eGFR: Estimated glomerular filtration rate
UA: Serum uric acid
PT: Prothrombin time
INR: International normalized ratio
APTT: Activated partial thromboplastin time
FIB: Fibrinogen
TT: Thrombin time
FDP: Fibrinogen degradation products
ESKD: End stage kidney disease
ACEI: Angiotensin-converting enzyme inhibitor
ARB: Angiotensin receptor blockers
