# Supplementary material for: Characteristics of renal pathology and coagulation function in IgA nephropathy and IgA vasculitis associated nephritis
Source: BMC Nephrol. 2024 Jan 25;25:35. doi: 10.1186/s12882-024-03465-6 (PMC10811929; doi:10.1186/s12882-024-03465-6)
Supplement: Supplementary file 1 — Supplementary Material 1 [file 12882_2024_3465_MOESM1_ESM.docx]

| Supplementary Table 1. Pathological data of IgAN patients based on Oxford classification. | | | | |
| --- | --- | --- | --- | --- |
| MEST-C score | | IgAN | IgAVN | *P* value |
| M | M0 | 73 (28.9%) | 13 (18.3%) | 0.075 |
|  | M1 | 180 (71.1%) | 58 (81.7%) |  |
|  |  |  |  |  |
| E | E0 | 223 (88.1%) | 57 (80.3%) | 0.088 |
|  | E1 | 30 (11.9%) | 14 (19.7%) |  |
|  |  |  |  |  |
| S | S0 | 79 (31.2%) | 48 (67.6%) | <0.001 |
|  | S1 | 174 (68.8%) | 23 (32.4%) |  |
|  |  |  |  |  |
| T | T0 | 213 (84.2%) | 69 (97.2%) | 0.014 |
|  | T1 | 29 (11.5%) | 2 (2.8%) |  |
|  | T2 | 11 (4.3%) | 0 |  |
|  |  |  |  |  |
| C | C0 | 192 (75.9%) | 44 (62%) | 0.065 |
|  | C1 | 57 (22.5%) | 25 (35.2%) |  |
|  | C2 | 4 (1.6%) | 2 (2.8%) |  |
| mesangial [M] and endocapillary [E] hypercellularity, segmental sclerosis [S], interstitial fibrosis/tubular atrophy [T], and crescents [C]. Categorical variables are presented as the number of patients (percentage). | | | | |
